# Progress and prospects in flexible tactile sensors

**DOI:** 10.3389/fbioe.2023.1264563

**Published:** 2023-09-26

**Authors:** Ya-Feng Liu, Wei Wang, Xu-Fang Chen

**Affiliations:** ^1^ College of Artificial Intelligence, Southwest University, Chongqing, China; ^2^ College of Aerospace Engineering, Chongqing University, Chongqing, China; ^3^ Chongqing 2D Materials Institute, Chongqing, China

**Keywords:** flexible tactile sensors, stretchable materials, human signal monitoring, robotic, human–computer interaction

## Abstract

Flexible tactile sensors have the advantages of large deformation detection, high fault tolerance, and excellent conformability, which enable conformal integration onto the complex surface of human skin for long-term bio-signal monitoring. The breakthrough of flexible tactile sensors rather than conventional tactile sensors greatly expanded application scenarios. Flexible tactile sensors are applied in fields including not only intelligent wearable devices for gaming but also electronic skins, disease diagnosis devices, health monitoring devices, intelligent neck pillows, and intelligent massage devices in the medical field; intelligent bracelets and metaverse gloves in the consumer field; as well as even brain–computer interfaces. Therefore, it is necessary to provide an overview of the current technological level and future development of flexible tactile sensors to ease and expedite their deployment and to make the critical transition from the laboratory to the market. This paper discusses the materials and preparation technologies of flexible tactile sensors, summarizing various applications in human signal monitoring, robotic tactile sensing, and human–machine interaction. Finally, the current challenges on flexible tactile sensors are also briefly discussed, providing some prospects for future directions.

## 1 Introduction

Flexible tactile sensors have attracted significant attention recently owing to their comfortable interface and long-term continuous signal acquisition capabilities (Guo et al., 2017; Li G. et al., 2020; Bae et al., 2022; Chen et al., 2023). Flexible tactile sensors can be integrated onto complex surfaces such as human skin or soft robots due to their low modulus, stretchability, and light weight (Pang et al., 2020; Ainsworth et al., 2022; Liu et al., 2023). Therefore, materials and fabrication techniques for flexible tactile sensors have received significant attention from researchers. Material innovation is the key to achieving flexibility and stretchability of tactile sensors (Jang et al., 2016). Currently, the materials used for flexible tactile sensors mainly include metal, conductive polymer, and composite materials (Castellanos-Ramos et al., 2010; Yoshimura et al., 2016; Kim et al., 2021; Mondal et al., 2022; Mohan et al., 2023). Moreover, traditional fabrication methods cannot meet the needs of complex structure manufacturing and highly personalized monitoring of humans (Ainsworth et al., 2022). A novel advanced manufacturing technology has been developed for flexible tactile sensors, mainly including 3D printing, screen printing, direct ink writing, inkjet printing, pattern transferring processes, spray/spin coating, drop casting, and vacuum filtration processes (Wen et al., 2003; Ishihara et al., 2006; Castellanos-Ramos et al., 2011; Sato et al., 2019; Zhang et al., 2021; Chen et al., 2022; Lee et al., 2022). With advances in materials, processes, and sensing technologies, various flexible tactile sensors have been developed for great potential applications (Amjadi et al., 2016). This paper discusses the materials and preparation technologies of flexible tactile sensors, summarizing various applications in human signal monitoring, robotic tactile sensing, and human–machine interaction. Finally, the current challenges on flexible tactile sensors are also briefly discussed, providing some prospects for future directions.

## 2 Materials

The development of flexible tactile sensors largely relies on advancements in material science (Cai and Wang, 2015). The materials for flexible tactile sensors are mainly classified as metal materials, polymer materials, and composite materials. Table 1 shows the properties of different materials.

**TABLE 1:** Properties of materials.

Material	Properties
Traditional metal	Ductility, thermal conductivity, electrical conductivity, and convenient production
Liquid metal	Outstanding stretchability and self-healing ability
Carbon black-based active materials	Low-cost and high electrical conductivity
CNT-based active materials	Excellent strength, superior conductivity, good optical transparency, and outstanding thermal stability
Graphene-based active materials	Superior electron mobility, excellent strength, and low resistance
Nanowire/nanoparticle (NW/NP)-based one-dimensional nanostructured materials	Outstanding electrical conductivity and excellent flexibility
Conductive polymer	Good conductivity and biocompatibility

### 2.1 Metal

Metals have widespread applications among sensing materials. Patterned structures and interconnect techniques for metal are reliable with the capability of obtaining high-resolution structures. Tactile sensors that are prepared on flexible substrates with metals could significantly shift human perceptions of electronics, providing potential applications in human signal monitoring, robotic tactile sensing, and human–machine interaction (Forrest, 2004).

#### 2.1.1 Traditional metals

**FIGURE 1:**
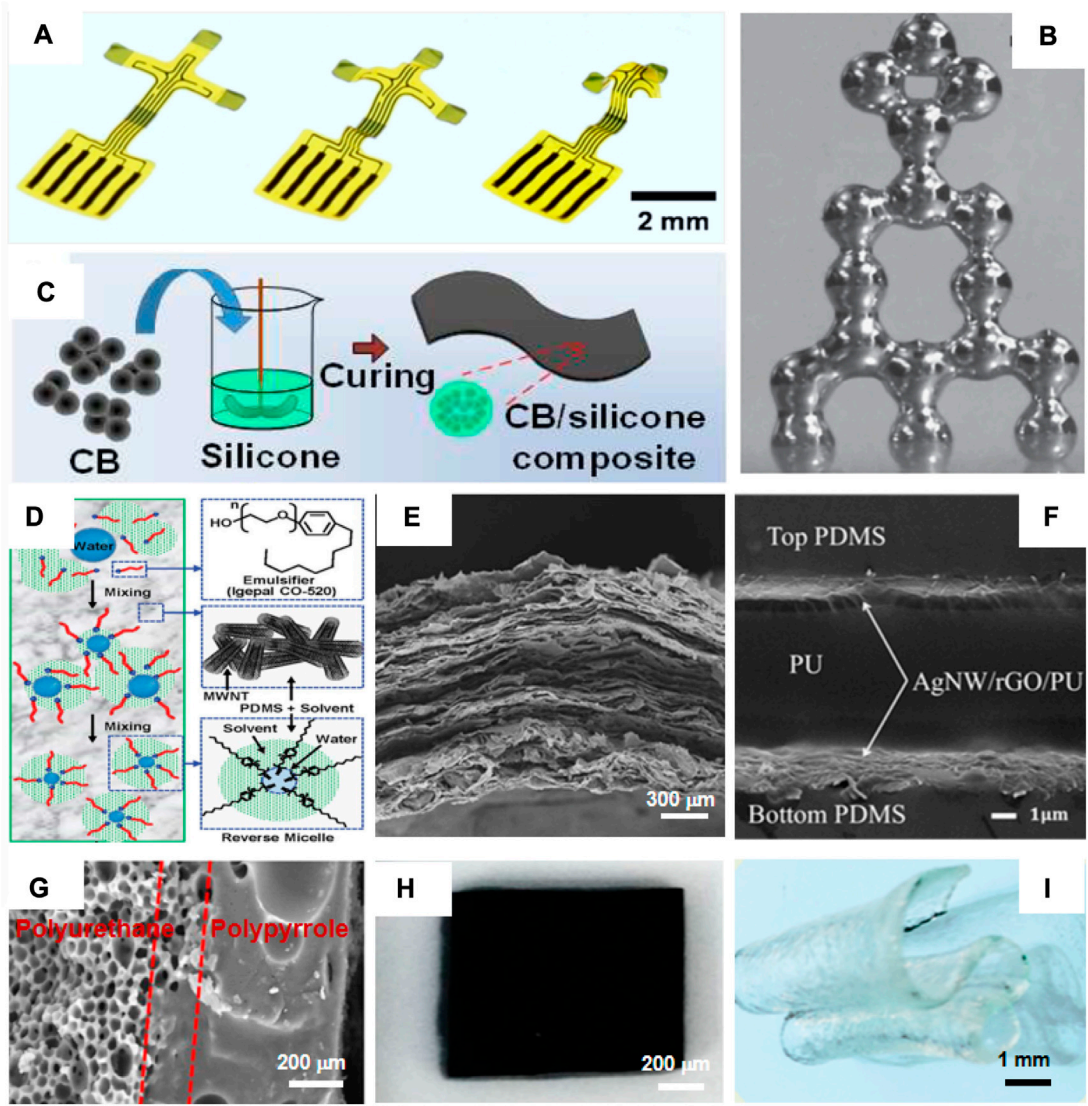
Materials for flexible tactile sensors. **(A)** Traditional metal, **(B)** liquid metal, **(C)** carbon black-based active materials, **(D)** CNT-based active materials, **(E)** graphene-based active materials, **(F)** nanowire-/nanoparticle-based one-dimensional nanostructured materials, **(G)** polyurethane/polypyrrole, **(H)** PEDOT: PSS, and **(I)** hydrogels (Ladd et al., 2013; Hou et al., 2014; Jung et al., 2014; Li et al., 2014; Lee et al., 2016; Choi et al., 2017; Won et al., 2019; Fu et al., 2020; Liu et al., 2020).

Traditional metals for flexible tactile sensors include gold (Au), silver (Ag), copper (Cu), magnesium (Mg), zinc (Zn), aluminum (Al), chromium (Cr), and titanium (Ti). The outer shell electrons of metals can move freely within the metal, which enables excellent electrical properties. The conductivity of metals can be adjusted by the length and diameter of the metal strain-sensitive grating. John A. Rogers et al. reported a flexible tactile sensor system based on Cr/Au that achieved ultra-thin thicknesses, compatible elastic modulus, and bending stiffness, matched to human skin (Figure 1A) (Won et al., 2019). The flexible tactile sensor on the skin achieves a conformal attachment and adequate adhesion, according to van der Waals interactions alone. It has a variable shape, which enables the mechanically guided geometric transformation of a two-dimensional planar precursor to generate a 3D structure capable of decoupling complex stress/strain. Complex stimulus responses such as pressure, shear, tension, and bending, which are difficult to achieve with traditional rigid sensors, are essential for soft robotics, human signal monitoring, and human interactions with the physical environment.

#### 2.1.2 Liquid metals

The development of liquid metals enriched the system of sensing materials. Liquid metals (Figure 1B), which are in the fluid phase at normal atmospheric temperature, have been widely used as conductors for flexible devices due to their outstanding stretchability and self-healing ability (Park et al., 2021). Gallium and mercury are typical liquid metals with low melting points. Gallium-based alloys have low volatility and negligible toxicity and hence are actively studied for application in flexible tactile sensors (Park et al., 2021). Gallium and gallium-based alloys exhibit remarkable electrical conductivity with excellent deformability, which provides the possibility of conformal attachment with human skin (Kim et al., 2019). Moreover, some alkali metals, such as rubidium, cesium, and francium, also exist in a fluid state at or near normal atmospheric temperature. The conductivity of liquid metals can be adjusted by the shape of the liquid metal. Inkyu Park et al. introduced a liquid metal-based flexible pressure sensor with a microbump for human signal monitoring. The liquid metal microchannel and rigid microbump arrays are fabricated using the one-step direct process. The sensitivity of the flexible pressure sensor is approximately 0.158 kPa^−1^, with a repeatable pressure response and ignorable hysteresis under fatigue stimulus (Kim et al., 2019). In recent years, various flexible tactile sensor-based liquid metals have been developed for long-term monitoring of human signals in disease diagnosis (Liu L. et al., 2022).

### 2.2 Composites

In recent years, micro-/nanomaterial-based composite materials have attracted considerable research interest in flexible electronics by reasons of excellent carrier mobility, flexibility, and low cost, holding great promise for high-performance flexible tactile sensors. Micro/nano conductive materials mainly include graphene, graphene oxide (GO), carbon nanotubes (CNTs), carbon black (CB), and nanowires/nanoparticles (NW/NPs), which are commonly mixed with polymers to produce conductive composites.

#### 2.2.1 Carbon black-based active materials

CB (Figure 1C) is a type of paracrystalline carbon comprising spherical particles with significant carbon layer ordering, with the size ranging from tens to a few hundred nanometers. There are honeycomb-like micropores inside carbon black. Electrons can move in micropores, forming conductive pathways. The conductivity of carbon black-based active materials is significantly enhanced after the addition of carbon black to the matrix (Fu et al., 2020). The combination of low-cost production and high electrical conductivity allows CB to be considered an essential component of conductive networks. Wu et al. designed a multi-functional pressure sensor based on a carbon black sponge with a micro-crack design, which can meet the requirements of ultra-small and large-scale motion monitoring through layer-by-layer assembly (Wu et al., 2016). However, most of the dangling bonds at the edges of the carbon layer are bonded to hydrogen bonds. The conjugated structure of the carbon layer is disrupted, resulting in a decrease in conductivity, causing higher electric potential for flexible tactile sensors to operate.

#### 2.2.2 CNT-based active materials

CNT (Figure 1D) is a one-dimensional carbon nanomaterial with excellent strength, superior conductivity, good optical transparency, and outstanding thermal stability. It can be synthesized through various methods, such as chemical vapor deposition, laser ablation, and arc discharge. A single nanotube shows superior conductivity, with the current levels of up to 25 μA per tube (Wang et al., 2013). A nanofiber/nanotube aerogel tactile sensor was developed by cellulose nanofibers with excellent mechanical properties, which are functionalized by small walled carbon nanotubes. Carbon nanotubes form conductive networks inside the nanofiber/nanotube aerogel. They exhibit an elastic mechanical behavior, which combines with a reversible electrical response under compression to achieve responsive conductivity and pressure sensing. A pressure change of 0.1 bar causes a relative change of 10% in resistance. The electrical properties of CNTs play an important role in the performance of CNT-based active materials, which depend on the type, size, and orientation of CNTs in the polymer matrix.

#### 2.2.3 Graphene-based active materials

The superior electron mobility, excellent strength, and low resistance of graphene are attributed to the two-dimensional structure of carbon atoms with a honeycomb lattice. The laminated configuration enables adjacent overlapping graphene layers (Figure 1E) to shift the overlapping region by reversible sliding (Liu et al., 2016). It can be selected as building blocks, which are then assembled into graphene-based active materials. Liu et al. reported a high-performance strain sensor with a fish-scale graphene sensing layer, which is manufactured by stretching/releasing a composite film of reduced graphene oxide and elastic bands. It can be used to detect stretching and bending deformation with a wide sensing range, high sensitivity, and ultra-low detection limit (Liu et al., 2016). The laminated configuration enables adjacent overlapping graphene layers (Figure 1E) to shift the overlapping region by reversible sliding (Liu et al., 2016). In addition, graphene oxide (GO) is electrically insulated. It can have the functions of electric conductivity after being reduced by employing a chemical method or thermal method.

#### 2.2.4 One-dimensional nanostructured materials

One-dimensional nanostructured materials **(**such as nanowires**)** are provided with outstanding electrical conductivity and excellent flexibility and hence are expected to be competitive materials in the flexible tactile sensor (Akter and Kim, 2012; Wei et al., 2016). The good optical transparency of nanowires even achieves a breakthrough in the transparency of flexible tactile sensors. Apart from nanowires, nanoparticles have also been considered for the construction of conductive networks via a variety of methods (screen-printing or inkjet printing) because of their low cost, eco-friendly nature, and scalability. In addition, nanowire-/nanoparticle-based one-dimensional nanostructured materials (Figure 1F) can integrate the advantages and disadvantages of each material by combining two or more different materials, indicating significant latent capacity in the construction of conductive networks for flexible tactile sensors. Shu Gong et al. developed a high-sensitivity tactile sensor by sandwiching ultra-thin gold nanowire-impregnated paper between two thin polydimethylsiloxane sheets, which can be easily integrated and patterned in a large area to draw a spatial pressure distribution map (Gong et al., 2014). It is provided with great expectation owing to not only excellent mechanical and electrical performances, great stability, and remarkable human compatibility but also the ease of process by uncomplicated and cost-effective approaches.

### 2.3 Conductive polymers

Polypyrrole (Figure 1G) is a promising conductive polymer that has attracted widespread attention on account of its ease of production, good conductivity, biocompatibility, and great adhesion to a variety of materials. The good conductivity of polypyrrole is credited to an electron transfer along the conjugated π-molecular orbital skeleton and the movement of charge carriers (Li et al., 2014). In addition, PEDOT-based materials are another competitive material for flexible tactile sensors because of their outstanding thermal stability, great transparency, and adjustable conductivity (10^−4^–10 (Guo et al., 2017) S*cm^−1^). Commercially available conductive polymers (CPs) of poly (3,4-ethylenedioxythiophene)/poly (styrenesulfonate) (PEDOT:PSS) (Figure 1H) are applied to various well-known flexible tactile sensors. It is easily handled by traditional common methods owing to its solubility in water, forming uniform slurries with a flexible matrix. Furthermore, hydrogels (Figure 1I) exhibit excellent properties in terms of electricity, mechanics, optics, and swelling/expansion features (Lin et al., 2020; Xia et al., 2021). Hydrogels have a three-dimensional network of polymers and abundant water. They possess a high degree of flexibility due to the abundant water content (Cui et al., 2018) and recently attracted significant attention owing to their potential application prospects as an impeccable material for flexible tactile sensors (Wirthl et al., 2017; Wallin et al., 2018). Guo et al. developed a tactile sensor based on the poly (amidoxime)/polyethyleneimine hydrogel by a hydrogen bonding interaction, which has broad application prospects in wearable sensing devices (Guo X. et al., 2023).

## 3 Processing/fabrication methods

In recent years, as traditional fabrication techniques cannot agree with the personalized needs of people, various advanced manufacturing technologies have been used to manufacture physical objects, resulting in extreme and complex structures. Current methods to pattern flexible tactile sensors include photolithography, transfer printing, 3D printing, screen-printing, direct writing, inkjet printing, pattern transferring processes, spray/spin coating, drop casting and vacuum filtration processes, and solution deposition methods. In this section, the pros and cons of fabrication methods used for flexible tactile sensors are discussed. Table 2 shows the advantages and disadvantages of different preparation methods.

**TABLE 2:** Advantages and disadvantages of different preparation methods.

Method	Advantage	Disadvantage
Photolithography	High consistency and excellent precision	High cost
Transfer printing	Manufacturing on complex surfaces	Inefficiency
3D printing	Customizable and low cost	Low precision
Screen printing	High efficiency and simplicity	Low precision
Inkjet printing	Low cost	Low precision and nozzle blockage
Spray/spin coating	Simple and low cost	Low accuracy and low reliability
Pattern transfer	High cost-effectiveness and time-effectiveness	Low precision
Drop-casting and vacuum filtration processes	Simple, low cost, and controllable	Low uniformity and limitation of large area coverage

### 3.1 Photolithography

**FIGURE 2:**
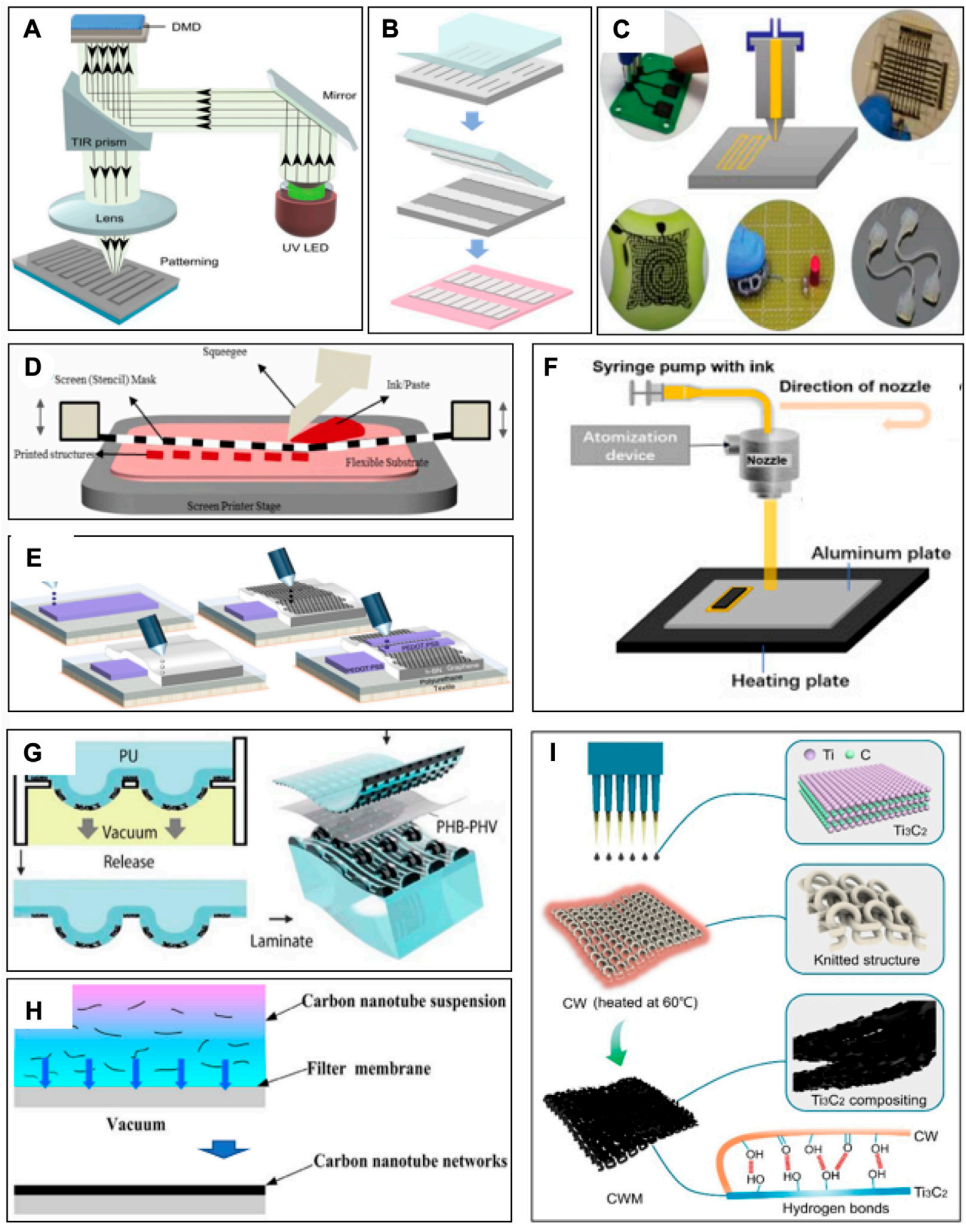
Processing/fabrication methods: **(A)** photolithography, **(B)** transfer printing, **(C)** 3D printing, **(D)** screen printing, **(E)** inkjet printing, **(F)** spray/spin coating, **(G)** pattern transferring, **(H)** vacuum filtration processes, and **(I)** drop casting (Mack et al., 2006; Song et al., 2009; Khan et al., 2015; Carey et al., 2017; Boutry et al., 2018; Liu et al., 2018; Abbasi et al., 2020; Chen et al., 2021; Guan et al., 2022).

Photolithography (Figure 2A) is the most common process for making patterns and topographical molds. Significant well-developed manufacturing and processing methods with high consistency and excellent precision make it a distinguished candidate for flexible tactile sensors as well. However, it has a high preparative cost.

### 3.2 Transfer printing

Transfer printing can be performed over large areas of uniform or segmented nanomembranes/ribbons using flexible structure stamps. It is usually used to obtain micro/nanopatterns on flat areas (such as wafers and glasses) and then transfer them onto flexible complex substrates with a flexible stamp. Figure 2B shows the processing programs involved in achieving the micro/nanopatterns by transfer printing. A conformable polydimethylsiloxane (PDMS) stamp is applied to pick up the free-standing microstructure silicon from the top of Si wafers and transfer it rapidly with controlled orientation to a flexible substrate (Mack et al., 2006). The type of adhesive stamping is significantly valuable for complex micro-structure processing.

### 3.3 3D printing

3D printing (Figure 2C) has been followed with interest, attributed to its simplicity in manufacturing, ability to customize microstructures, diverse types of printable materials, and low cost (Liu et al., 2018). 3D printing typically uses a heating nozzle to melt polymer filaments to fabricate objective models or a syringe nozzle to extrude printable ink with fitted rheological properties to fabricate flexible tactile sensors. A variety of printable materials can be modified with the required rheological performance, offering significant advantages for the manufacturing of flexible tactile sensors. This process provides excellent flexibility in the manufacture of enormously complex 3D flexible tactile sensors, which simulate the tactile perceptions of human skin to translate touch reception information such as tension, pressure, shear, torsion, bend, and vibration into electrical signals and play a significant effect on the application of flexible electronic skin (Frutiger et al., 2015; Yan et al., 2016).

### 3.4 Screen printing

The screen printer (Figure 2D) is provided with a sample structure including a screen, squeegee, press bed, and substrate. The screen printer is more generic than those of other methods due to the advantages of adaptability, affordability, and simplicity. The flexible tactile sensor can be developed quickly with several steps and can be reproduced by repeating the optimum operating procedure of screen printing. It should be pointed out that the printing quality and characteristics are influenced by a variety of factors, such as printing speed, solution viscosity, scraper angle, and geometric shape (Jabbour et al., 2001). Screen printing is inclined to high-viscosity inks. Without appropriate adjustments to ink performance and mesh size, a value of 50–100 µm is a common printing resolution with a thickness of several micrometers. The feasibility of screen printing for flexible tactile sensors is verified by a variety of flexible electronics devices (Chang et al., 2009). A variety of materials can be integrated into the screen-printed paste to enhance the sensing performance, which can be classified into three main classes, inorganic (Au, Ag, graphene, etc.), organic (polyaniline, polypyrrole, etc.), and composite (chitosan–Au nanocomposite, CNT-based composite, etc.). (Ong et al., 2021). Among a series of metals used for printed tactile sensors, Ag-based pastes are the most common material attribute for excellent electrical properties. In addition to silver solutions, carbon- and copper-based inks are often frequently selected in the preparation of flexible tactile sensors.

### 3.5 Inkjet printing

Inkjet printing (Figure 2E) is a high-speed patterning method for the direct patterning of liquid ink with low viscosities by a micrometer-sized inkjet nozzle. The droplets are sprayed with relevant pulses generated by the actuators, which enable specific patterns to be printed directly on the substrates with no masks. In addition, inkjet printing projects individual ink droplets from the nozzle to the desired position to avoid waste, making it a low-cost preparation procedure. Furthermore, inkjet printing is capable of producing a high-precision pattern, attributed to the small size of the droplets (Lakafosis et al., 2010). Zheng et al. show pictures of different patterned electronic devices fabricated by an inkjet printer on a flexible flat (Zheng et al., 2013). By increasing the electric field with the distance between the flexible substrate and the nozzle, the thickness of electronic devices within the nanometer range can be easily realized. It has been successfully applied to manufacture a series of flexible tactile sensors, in particular. The primary weakness of inkjet printing is that certain types of conductive inks are incompatible, attributed to large particle size and nozzle blockage.

### 3.6 Spray/spin coating

Spray/spin coating (Figure 2F) is a simple and low-cost method to fabricate flexible patterned films, which is one of the most general ways of extensive deposition (Lipomi et al., 2011; Wang et al., 2019). Nevertheless, the manufacturing accuracy is relatively low, attributed to airbrush, which increased preparing costs and reduced reliability. To overcome this difficulty, a spray coating method combining a digital x–y plotter and a heated substrate is created to manufacture a CNT-based pattern film (Wan et al., 2017). However, the CNT-based pattern film deposited by the spray coating needs to be a properly functionalized substrate to achieve excellent adhesion (Fu and Yu, 2014).

### 3.7 Pattern transfer

In the pattern transfer process (Figure 2G), pattern arrays were pre-formed on the substrate, and then, the functional materials formed the required structural array by means of reverse molding. The pattern transfer process is more cost-effective and time-effective than traditional photolithography in the realization of flexible tactile sensors.

### 3.8 Drop casting and vacuum filtration processes

Solution-based micro/nanomaterials can be easily coated onto the substrate and formed into patterns through the methods of drop casting (Figure 2H) and vacuum filtration (Figure 2I) (Jeong et al., 2015; Eslamian and Soltani-Kordshuli, 2018). Drop-casting is simple, low cost, and controllable compared with other coating methods (Huang J. F. et al., 2015; Hong et al., 2017). However, the uniformity of the film prepared by drop casting is quite low, attributed to the coffee-ring effect, accompanied by the limitation of large-scale coverage (Deegan et al., 1997). In order to upgrade the uniformity, Zhao et al. studied the interfacial influence between the substrate surface and the reduced graphene oxide. It is indicated that the catechol unit has an effect on the uniform reduced graphene oxide film on the flexible substrate. Therefore, functional modifications to materials are helpful in manufacturing flexible tactile sensors on flexible substrates using drop casting and vacuum filtration methods (Zhang et al., 2014; Zhao et al., 2014; Hossain et al., 2019).

## 4 Applications

Flexible tactile sensors have substantial potential applications. Low-strain flexible tactile sensors are able to monitor structure health, stress/strain, etc. Meanwhile, largely stretchable tactile sensors can be integrated with humans, attaching to clothing or directly laminating onto skin, for movement monitoring, ranging from small deformation caused by respiration and pulse to large deformation such as stretching, bending, and twisting of body joints. In addition, flexible tactile sensors might be good for stress monitoring in soft robots (Amjadi et al., 2016). They exhibit different working ranges and sensing performances, which made it possible to adapt our detection methods to different environments (Yao et al., 2015). A large number of application examples have proven the possibility of flexible tactile sensors in human signal monitoring, robotic tactile sensing, and human–computer interaction.

### 4.1 Human signal monitoring

#### 4.1.1 Health monitoring

**FIGURE 3:**
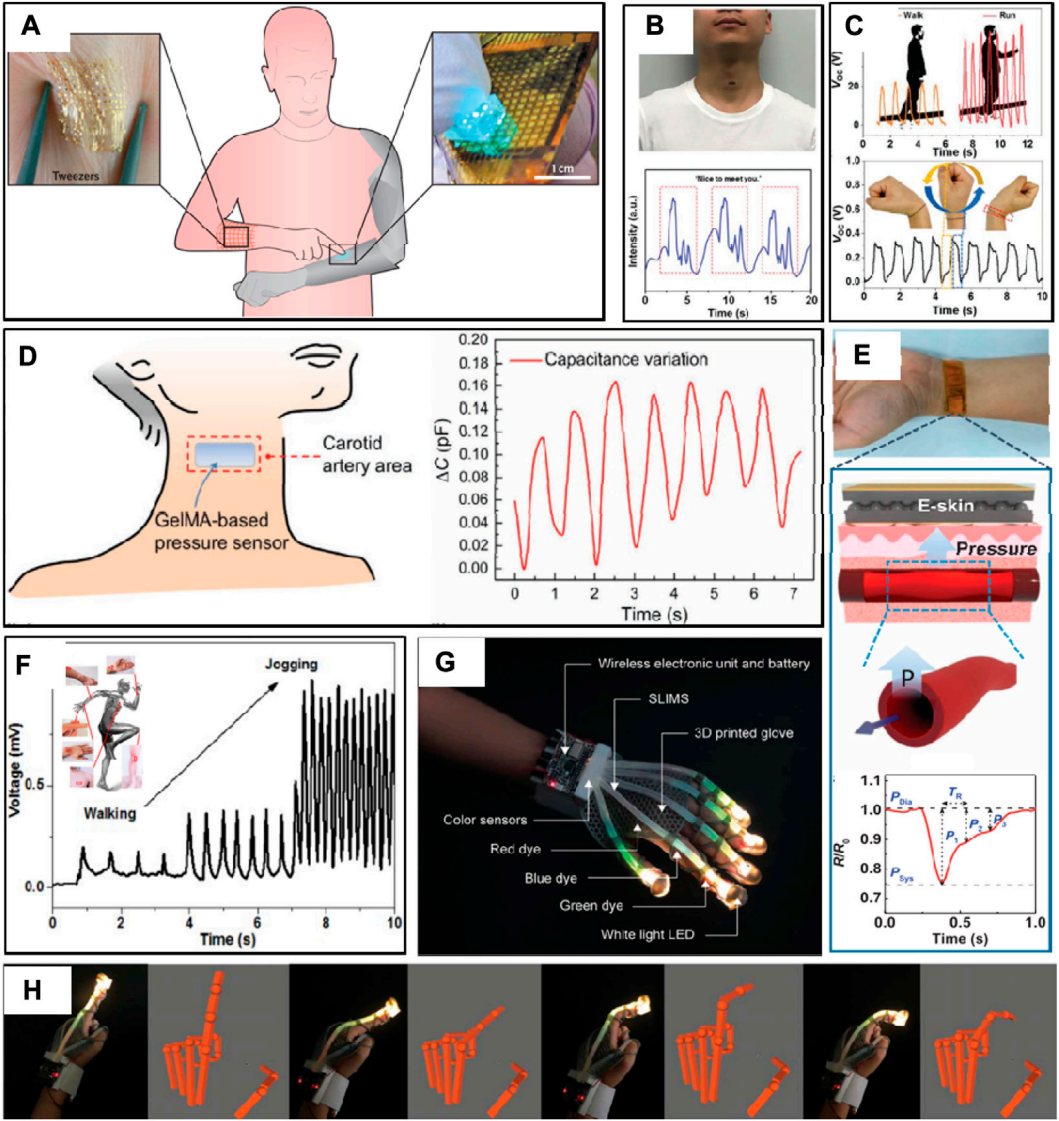
Human signal monitoring. **(A)** User-interactive touching; **(B)** swallowing; **(C)** twisting motion of the wrist; **(D)** vocal cord vibration detection; **(E)** artery pulse pressure; **(F)** human motions, including walking and jogging; **(G)** a flexible tactile sensor composed of parallel assemblies of elastomeric light guides; and **(H)** multilocation decoupling and multimodal deformation decoupling (Bauer, 2013; Park et al., 2015; Han et al., 2019; Bai et al., 2020; Li Z. et al., 2020; Ning et al., 2020).

With the increasing attack rate of chronic diseases such as hypertension and diabetes, an increasing number of people begin to follow medical care with interest. It is necessary for long-term monitoring of patients for the treatment of chronic diseases. However, traditional medical equipment is cumbersome, making it unsuitable for long-term monitoring. Flexible tactile sensors can be integrated into the wrist and chest to record blood pressure, pulse, and respiratory rate, which is highly related to human health (Figures 3A–E). For example, cellulose composite-based flexible tactile sensors with excellent sensitivity have shown outstanding performance in pulse recording (Chen S. et al., 2018) and respiratory rate (Chen Y. et al., 2018) detection. It exhibits precise peak changes and good stability under pressure (Huang H. D. et al., 2015; Guo et al., 2022a). In addition, a cost-efficient method has been put forward to manufacture multifunctional three-dimensional carbon nanofiber network-based flexible tactile sensors with excellent stress sensitivity using the electrospinning technique. The stress sensitivity of the carbon nanofiber network-based flexible tactile sensor is 1.41 kPa^−1^, (Han et al., 2019), which also shows repeatable elasticity, great flexibility, and significant compressibility (>95%). Vocal cord vibration, pulse fluctuations, lung breathing, and limb movements are detected by flexible tactile sensors (Li Z. et al., 2020). Overall, flexible tactile sensors will play a significant role in the human health monitoring field, attributed to the advantages of being light weight, having good elasticity, facilities for put on, and long-term monitoring.

#### 4.1.2 Motion monitoring

Flexible tactile sensors can be integrated into the skin of limbs to collect motion signals, which is conducive to evaluating the state of muscle contraction and relaxation. It can also be installed on the human joints to detect a variety of motion modes in real-time, such as walking, jumping, running, and squatting (Figure 3F). In addition, it is possible to evaluate the posture of athletes and adjust the exercise plan in a timely manner by feedback information to upgrade the training efficiency. A flexible tactile sensor is developed, which is made up of elastic light-guide parallel components (Figure 3G). The flexible tactile sensor can detect the position, size, and mode of mechanical deformation (stretching, bending, or pressing) (Wang et al., 2022; Guo X. H. et al., 2023). The flexible tactile sensor is integrated with a wireless glove, which demonstrates the applications of multi-position decoupling and multi-mode deformation decoupling (Figure 3H).

### 4.2 Robotic tactile sensing

**FIGURE 4:**
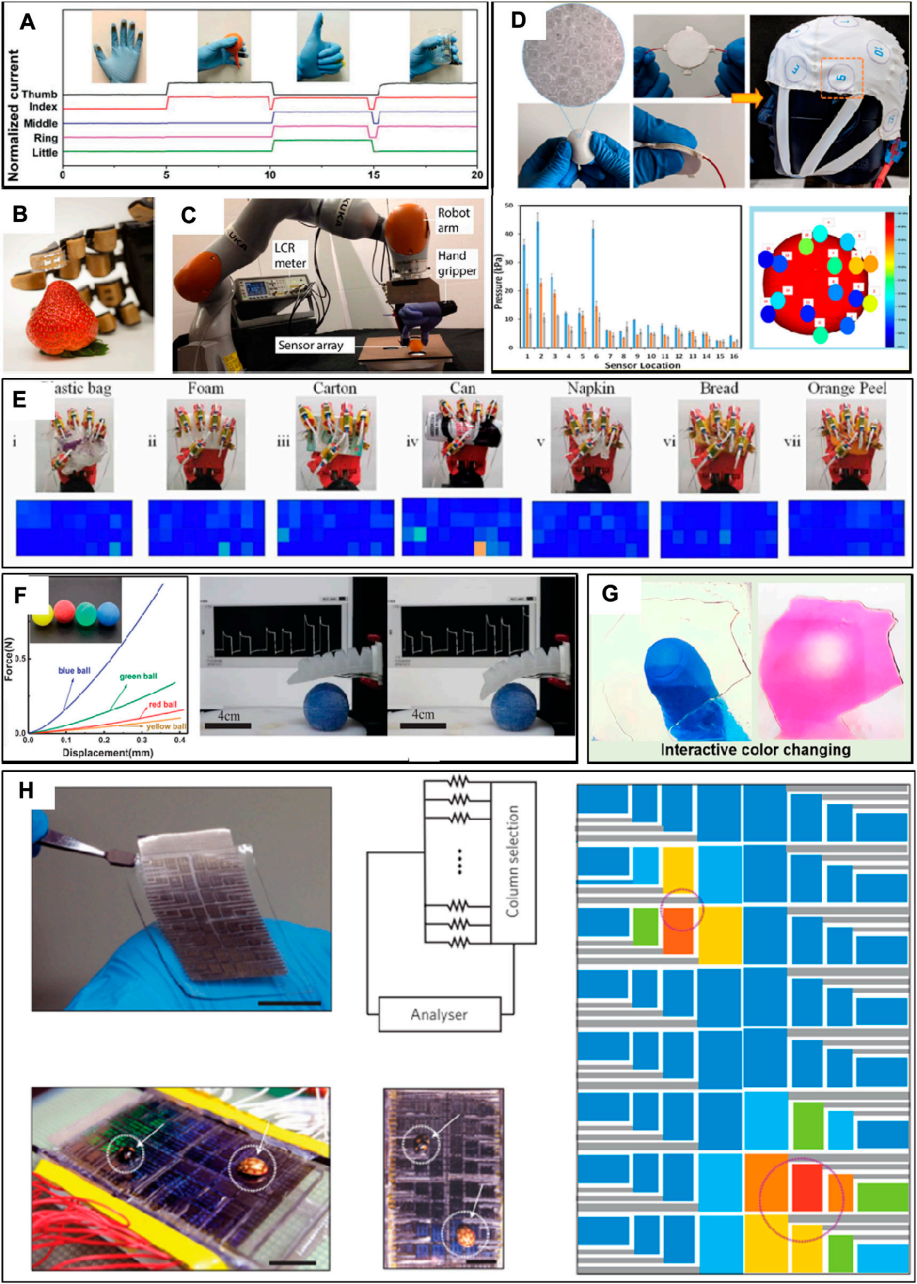
Environmental signal monitoring. **(A)** Responses to various hand motions. **(B)** A robotic hand with an integrated compliance sensor array touching a strawberry. **(C)** A flexible tactile sensor mounted on an artificial hand was exposed to shear force with a ping-pong ball. **(D)** Flexible tactile sensing fit cap demonstration. **(E)** A group of example signal maps when the robot hand grips seven types of garbage. **(F)** Softness detection. **(G)** Interactive color changes with pressure. **(H)** Fabrication of a sensor network to measure the spatial distribution of an input pressure signal (two lady beetles placed at two locations) (Pang et al., 2012; Boutry et al., 2018; Liang et al., 2019; Sun et al., 2019; Li G. et al., 2020; Beker et al., 2020; Liu et al., 2020; Masihi et al., 2021).

Tactile perception is necessary for precise operations of objects, which will provide crucial tactile perception for robots. Flexible tactile sensors can enhance the safety of object operation and the accuracy of object detection (Figure 4A). In addition, adaptation is a special characteristic of human skin. Attributed to the complexity of sensing mechanisms, only high-precision or bulky handheld instruments are applied to detect material compliance. Flexible tactile sensors are provided with compliance mapping, which can offer a human-like sensation for the robot system when grasping objects composed of multiple materials with varied compliance. It enables the robot to deal with a variety of sophisticated and complex missions on material classification (Figure 4B), normal force, and tangential force classification (Figure 4C). A novel flexible tactile sensor-based porous polydimethylsiloxane is fabricated with a wide pressure-sensing range by optimizing the porosity of the dielectric layer. A wearable fitness hat is manufactured based on 16 flexible tactile sensor arrays to monitor the pressure on the tester’s head (Figure 4D). The real-time spatial distribution of pressure is provided by the wearable fitness hat, which helps observe and understand the correct fit of the helmet in modern sports. Furthermore, a flexible tactile sensor enabling intelligent perception is prepared, which was integrated into the robotic surface to achieve a variety of complex operations (Figure 4E). It is indicated that the flexible tactile sensor is provided with the capability of classifying a variety of materials with excellent sensitivity (Figure 4F). By combining collected signals with machine learning, it can accurately identify different tasks, which shows a classification accuracy rate of 94%. In addition, a flexible tactile sensor-based electronic hat with multi-responsiveness was invented (Liu et al., 2019). The flexible tactile sensor, by fully mimicking human skin, can detect multiple signals of pressure, surface roughness, and airflow rate similar to those of human skin. Inspired by the structural color regulation capability of chameleons, a flexible tactile sensor is proposed for simultaneous tactile sensing and interactive color changing (Figure 4G). All of those flexible tactile sensors provided substantial information for operating a robot’s arm in a variety of missions, indicating prospective applications in robots with tactile feedback (Figure 4H).

### 4.3 Human–computer interaction

**FIGURE 5:**
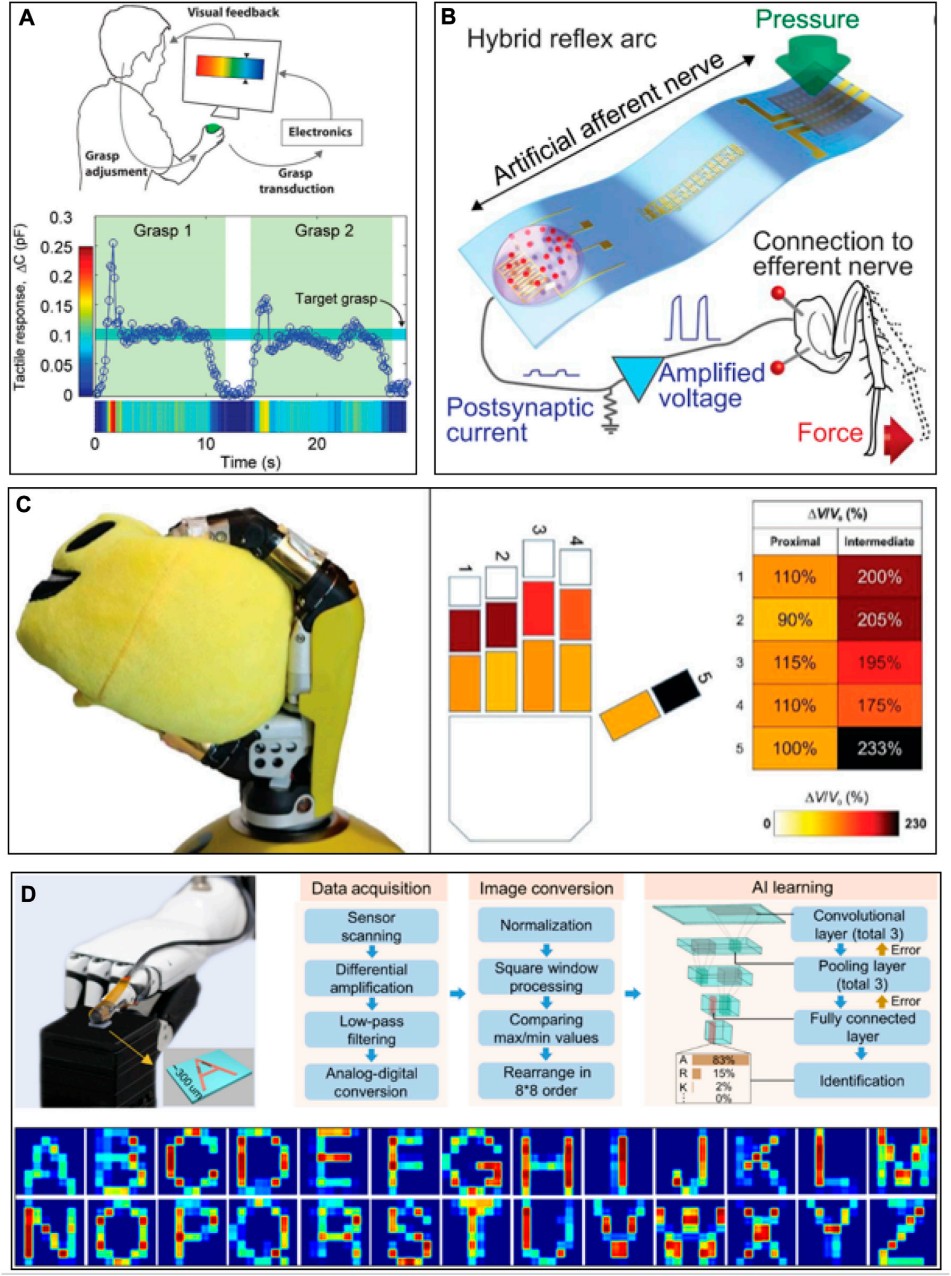
Human–computer interaction. **(A)** Tactile response during a grasp and manipulation task. **(B)** A hybrid reflex arc made of an artificial afferent nerve and a biological efferent nerve. **(C)** Grabbing of a soft ball, enabling tactile feedback. **(D)** A robotic finger with a microarray sensor is touching the letter patterns of collected letter images of A to Z through flow (Gerratt et al., 2015; Núñez et al., 2017; Kim et al., 2018; Pang et al., 2020).

Perception and interaction are the foundation to achieve communication between the virtual and physical worlds of humans and the environment (Figure 5). Conventional hard electronic devices cannot afford conformable attachment with the human skin or soft robots, which constrain information communication and interaction between the virtual and physical space (Ma et al., 2020). Currently, the main goal of researchers is to establish a smart human–machine interface system with flexible tactile sensors, which is used to obtain the physiological signals of humans to control intelligent robots or other objects (Guo et al., 2022b; Guo et al., 2022c). For example, a flexible strain sensor based on cellulose composites is invented to develop intelligent gloves. Physiological signals of bending fingers are obtained by the flexible tactile sensor, which controls intelligent robots with a controller and reduces the deviation with real-time feedback. It is indicated that intelligent gloves can perform surgery or some delicate and dangerous tasks that humans cannot complete (Figure 5A). In addition, flexible tactile sensors are used to mimic the functions of human sensory nerves (Figure 5B). Artificial afferent nerves collect stress signals from clusters of tactile sensors and convert the stress signal into action potentials using ring oscillators. Moreover, a flexible tactile microarray sensor-based graphene aerogel with ultrasensitivity and ultrastability was developed (Figure 5C), which achieved high accuracy (80%) in artificially intelligent touch identification that outperformed that of human fingers (30%). Due to its excellent sensitivity, low manufacturing cost, and flexibility, the intelligent system fabricated with flexible tactile sensors has the advantage of digital recognition over the conventional system (Figure 5D).

**FIGURE 6:**
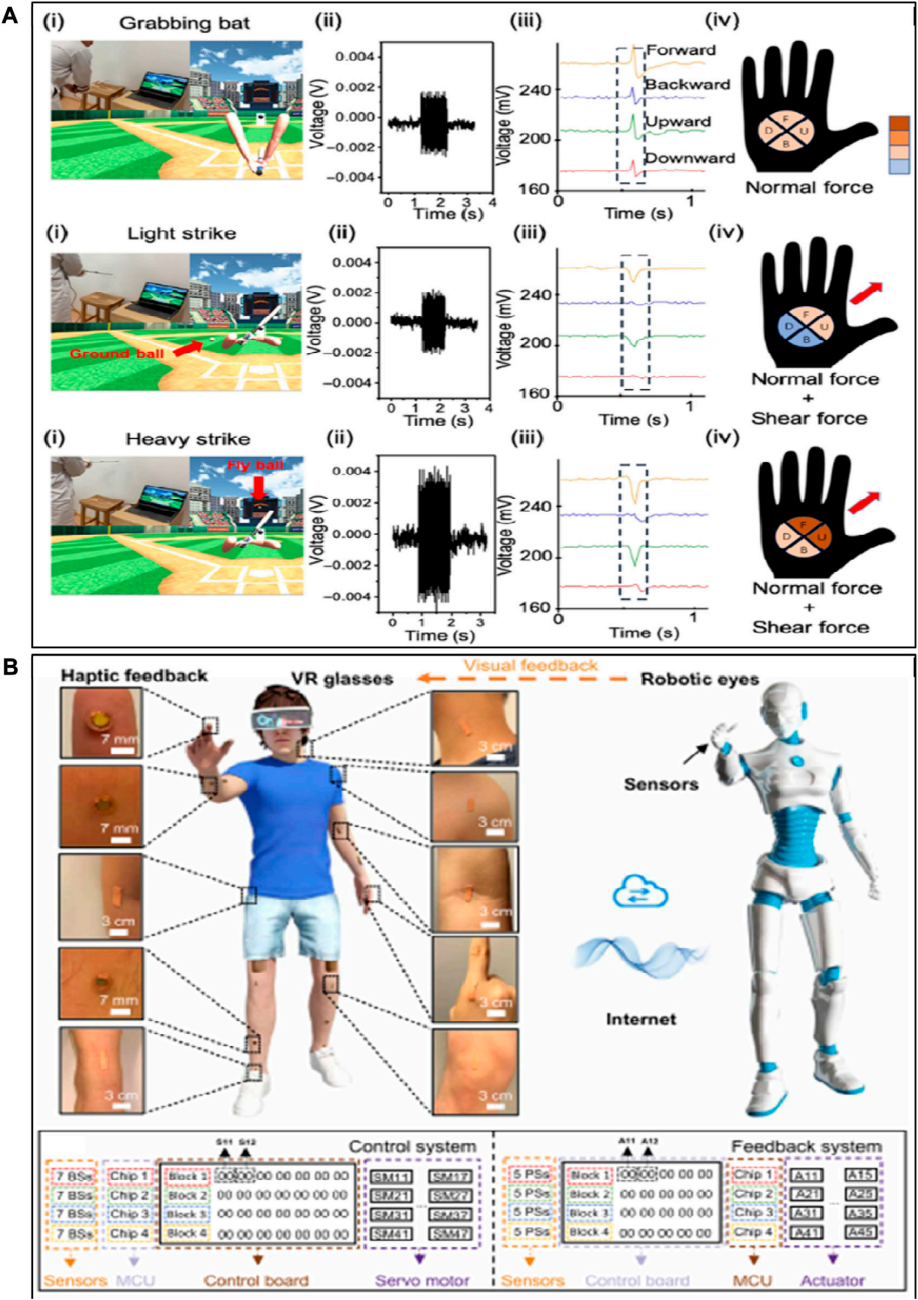
Human computer interaction. **(A)** Haptic-feedback smart glove as a creative human‒machine interface for virtual augmented reality applications and **(B)** electronic skin as wireless human‒machine interfaces for robotic VR (Zhu et al., 2020; Liu Y. M. et al., 2022)

The human–machine interface (HMI) provides opportunities for the interaction between humans and machines, contributing a significant effect in remotely operating robots. The traditional HMI based on bulky, rigid, and costly machines mainly concentrates on robot/machine control but lacks sufficient feedback to humans, which significantly constraints business applications in executing complex missions. With increasing demands for intuitive and effective manipulation, closed-loop HMIs with both precise sensing and feedback functions are essential. A tactile feedback intelligent glove with flexible tactile sensors has been proposed for the virtual reality system. In virtual space, electrical signals with different degrees of freedom on human hands are used to detect multi-directional bending and sliding states. Object detection can be realized by it with an accuracy rate of 96% (Figure 6A). In addition, a closed-loop HMI system based on flexible tactile sensors is also developed, which is compatible with the entire body and provides wireless movement capture and tactile feedback through the internet, wireless fidelity, and Bluetooth (Figure 6B). Closed-loop HMI-integrated tactile virtual reality shows tremendous potential in various fields such as entertainment, household healthcare, sports exercise, non-contact biological sample collection, and infectious disease patient care.

## 5 Conclusions and perspectives

Flexible tactile sensors have made important progress in materials and processing/fabrication methods. The essential features, such as excellent sensitivity, quick response, low cost, and stretchability/flexibility, have been significantly enhanced. Compared to traditional fabrication techniques such as photolithography, new fabrication techniques are provided with more cost-effective methods as they do not need costly masks or facilities. Using the direct deposition method, the time from design to production of flexible tactile sensors is saved, and the waste of materials is minimized. New preparation methods are provided to manufacture flexible tactile sensors with customized geometries to satisfy the dimensional requirements and to upgrade their properties. It is crucial to utilize those technological innovations to address the obstructions to manufacturing flexible tactile sensors. In summary, new preparation methods for the development of flexible/stretchable tactile sensors paved the way for compliant sensing systems that conform to the complex unstructured skin of humans/robots.

Various aspects of people’s lives are put forward to intelligence and intelligent systems. It is indicated that incorporating various flexible tactile sensors into new manufactures will broaden the scope of applications in the future. At present, researchers mainly focus on improving the sensitivity of flexible tactile sensors. However, a variety of factors including the complexity of the environment and compatibility with soft robots are overlooked. High integration, multifunctionality, and extensive flexible tactile sensor coverage will proceed to be one of the most important future challenges in the sight of hardware. In the process of physical interaction with the environment, it is necessary to apply a large number of flexible tactile sensors to obtain complex information on a large area. Furthermore, a tactile signal can be integrated with data from other sensors to upgrade recognition accuracy (Someya et al., 2004; Someya et al., 2005; Takei et al., 2010; Zang et al., 2015). Furthermore, it is necessary to develop computational methods that run near or within sensor networks to reduce redundant data transfer between sensing and processing units to effectively process such a large amount of data and reduce energy dissipation (Zhou et al., 2019). The new intelligent artificial systems may enable tactile sensing and memory functions as well as neuromorphic pre-processing with upgraded processing efficiency and a higher recognition rate in subsequent processing tasks (Zhou and Chai, 2020).

In terms of application, current human health signal monitoring is at the initial level of information recording, which is still a long way from intelligent diagnosis (Qiu et al., 2019; Wang et al., 2022). How to combine tactile signal monitoring with intelligent data processing methods to enable the integration of intelligent monitoring and diagnosis is an important development direction (Guo et al., 2022b; Guo et al., 2022d; Guo X. H. et al., 2023). Moreover, based on the natural advantages of combining with the human body, flexible tactile sensing technology can easily realize stress/strain sensing and feedback on the human skin interface, which greatly promotes the development of virtual reality technology. As a result, the combination of autonomous control algorithms and tactile data processing methods to achieve tactile dexterity is an important development trend (Xia et al., 2022). In addition, if the tactile signal can be directly transmitted to the nerve or even the brain, the human body’s direct perception of the external world would be realized, and the human tactile signal would be extended to biology or external equipment. Therefore, the combination of flexible sensing technology and biotechnology has important development potential in the future.
